# Deletion of galectin-3 exacerbates microglial activation and accelerates disease progression and demise in a SOD1^G93A^ mouse model of amyotrophic lateral sclerosis

**DOI:** 10.1002/brb3.75

**Published:** 2012-07-23

**Authors:** Bruce J Lerman, Eric P Hoffman, Margaret L Sutherland, Khaled Bouri, Daniel K Hsu, Fu-Tong Liu, Jeffrey D Rothstein, Susan M Knoblach

**Affiliations:** 1 Department of Pharmacology, George Washington University School of Medicine and Health SciencesWashington, DC; 2 Department of Integrative Systems Biology, George Washington University School of Medicine and Health SciencesWashington, DC; 3 Department of Pediatrics and Center for Genetic Medicine, Children's National Medical CenterWashington, DC; 4 NINDS, Neurodegeneration Cluster, National Institutes of HealthRockville, Maryland; 5 Department of Dermatology, University of California Davis School of MedicineSacramento, California; 6 Department of Neurology, Johns Hopkins UniversityBaltimore, Maryland

**Keywords:** Alternative activation, amyotrophic lateral sclerosis, microglia, motor neuron disease, SOD1

## Abstract

Galectins are pleiotropic carbohydrate-binding lectins involved in inflammation, growth/differentiation, and tissue remodeling. The functional role of galectins in amyotrophic lateral sclerosis (ALS) is unknown. Expression studies revealed increases in galectin-1 mRNA and protein in spinal cords from SOD1^G93A^ mice, and in galectin-3 and -9 mRNAs and proteins in spinal cords of both SOD1^G93A^ mice and sporadic ALS patients. As the increase in galectin-3 appeared in early presymptomatic stages and increased progressively through to end stage of disease in the mouse, it was selected for additional study, where it was found to be mainly expressed by microglia. Galectin-3 antagonists are not selective and do not readily cross the blood–brain barrier; therefore, we generated SOD1^G93A^/Gal-3^−/−^ transgenic mice to evaluate galectin-3 deletion in a widely used mouse model of ALS. Disease progression, neurological symptoms, survival, and inflammation were assessed to determine the effect of galectin-3 deletion on the SOD1^G93A^ disease phenotype. Galectin-3 deletion did not change disease onset, but resulted in more rapid progression through functionally defined disease stages, more severely impaired neurological symptoms at all stages of disease, and expiration, on average, 25 days earlier than SOD1^G93A^/Gal-3^+/+^ cohorts. In addition, microglial staining, as well as TNF-α, and oxidative injury were increased in SOD1^G93A^/Gal-3^−/−^ mice compared with SOD1^G93A^/Gal-3^+/+^ cohorts. These data support an important functional role for microglial galectin-3 in neuroinflammation during chronic neurodegenerative disease. We suggest that elevations in galectin-3 by microglia as disease progresses may represent a protective, anti-inflammatory innate immune response to chronic motor neuron degeneration.

## Introduction

Amyotrophic lateral sclerosis (ALS) is heterogeneous in phenotype and genotype, and despite intense research effort, the underlying cause(s) remain obscure. Sporadic and familial forms of ALS share common pathophysiological features, including a marked neuroinflammatory response, characterized by glial activation and innate and adaptive inflammatory components (for reviews, see Ilieva et al. 2009 and Appel et al. 2011). In murine models of ALS, activated astrocytes and microglia are observed in neuroinflammatory foci in the spinal cord prior to onset of symptoms, and such areas correlate with pronounced regional motor neuron loss (Shibata et al. 1996). Infiltration of CD8+ T-suppressor/cytotoxic and CD4+ T helper cells is also prominent (Kawamata et al. 1992). These cells alter disease progression both independently and through apparent cross-talk with microglia (Kipnis et al. 2004; Beers et al. 2008). Generally, glia secrete soluble factors that may be toxic (reactive oxygen species, proinflammatory cytokines) or protective (growth factors), depending on local environment (Li et al. 2007). However, in models of ALS, transgenic expression of mutant hSOD1 in astrocytes and microglia results in glial phenotypes that are inherently neurotoxic compared to their wild-type counterparts (Boillee et al. 2006; Nagai et al. 2007). Thus, chronic neurodegeneration in ALS may evolve as a so-called noncell-autonomous process (Lobsiger and Cleveland 2007) that, in part, reflects a toxic glial microenvironment.

Galectins are multifunctional immunomodulatory lectins that are expressed by a variety of cells during normal development and in inflammatory or autoimmune diseases and cancer (reviewed in Norling et al. 2009). Recent observations that galectins are increased after acute CNS injuries and during chronic neurodegeneration (Byrnes et al. 2006; Zhou et al. 2010) may support a potential role for galectins in the adaptive immune response to the “threat” of cell stress, damage, or death, and potentially, in the repair function(s) of innate immunity (Kono and Rock 2008). Galectins contain a conserved carbohydrate recognition domain (CRD) with affinity for β-galactosides. They are secreted via a nonclassical pathway, but also found in intracellular compartments (Gong et al. 1999; Hughes 1999). Extracellularly, galectins form cross-links with cell surface glycoconjugates or the extracellular matrix, where they preferentially bind *N*-acetyllactosamine (Galß13GlcNAc or Galß1-4GlcNAC) residues. Because many cell surface receptors, including those for cytokines, growth factors, and neurotransmitters, contain glycoconjugates, galectins may modulate transmembrane signaling events that affect a variety of cell functions (Boscher et al. 2011).

Recently, expression profiling revealed increases in mRNA for several galectins within the spinal cord during late-stage disease in mouse models of ALS (Ferraiuolo et al. 2007; Gonzalez de Aguilar et al. 2008; Zhou et al. 2010). Expression of galectin-3, also known as Mac-2, correlates with microglial activation subsequent to neuronal degeneration in mouse models of ALS (Yamanaka et al. 2008; Saxena et al., 2009; Hossiani et al. 2011), but nevertheless, its role in the disease is unclear.

In this study, we initially assessed expression of multiple galectins, during chronic motor neuron disease in the SOD1^G93A^ mouse and in patients with sporadic ALS. As galectin-3 was the only galectin to progressively increase from early to late stage of disease in the mouse, and was also confirmed in patients with sporadic ALS, we then focused on the role of galectin-3 in disease progression. Currently, available galectin-3 antagonists are carbohydrate based and are neither particularly selective, nor able to cross the blood–brain barrier significantly (Pieters 2006). Therefore, we generated C57BL6 SOD1^G93A^/Gal-3^−/−^ knock-out transgenic mice for our studies.

## Materials and Methods

### Animals

Animal procedures were performed following National Institutes of Health Guidelines for Animal Use and Welfare and supported by an approved institutional animal protocol. B6SJL (stock no. 002726) or C57BL6 (stock no. 004435) mice with the G93A human SOD1 mutation (SOD1^G93A^; Gurney et al. 1994) were obtained from Jackson Laboratories (Bar Harbor, ME). For all studies, data were collected only from male mice, to avoid confounding issues of disease progression due to sex. The B6SJL strain was used for the expression studies. To avoid confounding background effects, the SOD1^G93A^/Gal-3^−/−^ knock-out was created on the C57BL6 SOD1^G93A^ strain, rather than B6SJL, as the founder galectin-3 knock-outs were on the C57BL6 background (Hsu et al. 2000). Female C57BL6 galectin-3 knock-out mice (Gal-3^−/−^; Hsu et al. 2000) were bred with nonlittermate transgenic C57BL6 SOD1^G93A^ males to yield homozygous C57BL6 SOD1^G93A^/Gal-3^−/−^ mice at the F2 generation. Transgenic offspring were genotyped by PCR amplification from tail tissue DNA. Briefly, tail clips were digested (12 h, 55°C) in lysis buffer (1 m Tris, pH8.8, 0.5 m EDTA, 10% Tween 20, 200 μg/mL Proteinase K), boiled (5 min) to inactivate Proteinase K, and centrifuged at 16,500 × *g* (2 min). PCR lysis buffer was combined directly with PCR reaction buffer (1X Flexi Buffer, 25 mm MgCl_2_, 10 mm of PCR nucleotide mix), primers, GoTaq DNA polymerase, and nuclease free water in a 50 μL reaction mixture. RT-PCR was used to amplify mutated SOD1 and disrupted galectin-3, and results visualized on 2% ethidium bromide agarose gels. Primers used to identify the human *mSOD1*^*G93A*^ gene were 5′-CATCAGCCCTAATCCATCTGA-3′ (forward) and 5′-CGCGACTAACAATCAAAGTGA-3′ (reverse). GaI-3^−/−^ mice were originally produced by interrupting the region coding for the CRD in exon 5, by inserting a neomycin resistant gene in a short intro 4-exon 5 segment (0.5 kb) (Hsu et al. 2000). Primers to identify galectin-3 deficient mice were 5′GTAGGTGAGAGTCACAAGCTGGAGGCC-3′ (binding upstream of intron) and 5′GTAGGTGAGAGTCACAAGCTGGAGGCC-3′ (binding upstream of the Neo cassette) and 5′CACTCTCAAAGGGGAAGGCTGACTGTC-3′ (binding common downstream sequence of exon). These primers amplify a 450-bp fragment in gal-3^+/+^ mice, a 300-bp fragment in gal-3^−/−^ mice, and both 450- and 300-bp fragments in gal-3^+/−^ heterozygotes.

### Human postmortem spinal cord tissue

Spinal cords from patients with sporadic ALS (*n =* 5) or from those who died from other causes (*n =* 4) were obtained from a postmortem tissue bank (Johns Hopkins University). Human samples were evaluated in accordance with HIPPA regulations and supported by approved IRB protocols at Johns Hopkins and Children's National Medical Center.

### RNA preparation and microarray

Lumbar spinal cords from male B6SJL/J SOD1^G93A^ transgenic and wild-type mice were isolated at 28, 42, 56, 70, 98, 112, and 126 days of age (*n =* 3 per group), extracted in Trizol (Life Technologies, Grand Island, NY), cleaned with RNeasy mini-columns (Invitrogen, Carlsbad, CA), quantified with a spectrophotometer, and assessed for quality by gel electrophoresis. RNA was considered to be of suitable quality when intact 28S and 18S ribosomal bands were visualizable upon ethidium bromide staining of samples resolved on a 1% agarose gel. Total RNA was amplified and synthesized as biotin-conjugated cRNA, fragmented, and hybridized to Mouse 430 2.0 Affymetrix arrays using reagents and methods supplied by the manufacturer (Affymetrix, Santa Clara, CA). Microarray data are publicly available at NCBI GEO (accession GSE18597). For RT-PCR, cDNA was synthesized from total RNA with the SuperScript III First-Strand synthesis system (Invitrogen) and subjected to Taqman RT-PCR on a ABI Prism 7900HT (Life Technologies). Galectin-3 DNA primer sequ-ences were forward-CGGTCGTAGGTGAGCATCGTTGAC[FAM]G and reverse-CCCTTTGAGAGTGGCAAACCAT. Samples (*n =* 3 per group) were normalized to the relative amounts of reverse transcribed GAPDH, and expression levels calculated using 2.2 Sequence Detection Software (all from Applied Biosystems, Foster City, CA).

### Western blot, TNF-α, and protein carbonyl assays

Spinal cord homogenates (*n =* 3 per group for Western blot, *n =* 4 per genotype for TNF-α and carbonyl assays) were prepared in Mammalian Protein Extraction Reagent (M-PER) buffer with protease inhibitors (Pierce Biotechnology, Rockford, IL) and protein concentrations determined with a BCA protein assay kit (Pierce Biotechnology). Proteins (15 μg) were resolved on 10% *tris*-glycine or *bis*-tris polyacrylamide gels and electrotransferred to Hybond ECL membranes, as previously described (Knoblach et al. 2004). Blots were blocked in blocking buffer (0.05% Tween 20, 5% milk powder in PBS) for 1 h, incubated with primary antibody for 1 h, washed 3× in 0.05% Tween in PBS, and then incubated with anti-HRP conjugated secondary antibody for 1 h. Blots were again washed 3× in 0.05% Tween in PBS, and then developed with an ECL chemiluminescent detection kit (Amersham, Piscataway, NJ) and exposed to film. Primary antibodies included goat anti-mouse or anti-human galectin(s)-1, -3, and -9 (1:500; R&D systems, Minneapolis, MN) and antiactin (1:5000; Sigma–Aldrich, St. Louis, MO). Secondary antibodies included horseradish peroxidase-conjugated goat anti-mouse or anti-rabbit (1:3000; Bio-Rad, Hercules, CA), or horseradish peroxidase-conjugated donkey anti-goat (1:3000; Santa Cruz Biotechnology, Santa Cruz, CA). For TNF-α, total protein (50 or 150 μg) was assessed in triplicate using a Multi-Analyte ELISA kit (SABiosciences, Rockville, MD). Protein carbonyls were detected with an OxiSelect Protein Carbonyl ELISA Kit (Cell Biolabs, San Diego, CA). Absorbance was read at 450 nm.

### Immunohistochemistry

Animals were perfused with PBS and 4% paraformaldehyde and spinal cords were removed, cryoprotected, frozen, and cut (20 μm). Sections (animal or human) were fixed in 4% paraformaldehyde (10 min), washed in PBS, and blocked in 5% donkey serum in 0.1% Triton X-100/PBS (1 h). Primary antibodies were applied (2 h, room temperature), sections were then washed in PBS, incubated with secondary antibody (1 h, room temperature), washed again, cover slipped, and viewed with a Bio-Rad MRC1024 confocal fluorescent microscope. Primary antibodies were as follows: goat anti-galectin-3 (1:500; R&D Systems), rabbit anti-GFAP (astrocyte marker; 1:1000; Sigma-Aldrich), and rabbit anti-IBA1 (microglial marker; 1:2000; Wako Chemical, Richmond, VA). These antibodies cross react with both mouse and human. Alexa 488 donkey anti-goat (1:2000; Jackson Immunoresearch Labs, West Grove, PA) and Alexa 568 donkey anti-rabbit (1:2000; Invitrogen, Grand Island, NY) were used as secondary antibodies. For Nissl staining, Alexa Fluor FITC-conjugated Nissl (1:5000; Invitrogen) was applied to sections (10 min), and thereafter, sections were again rinsed in PBS.

### Survival and neurological function

Animals (*n =* 12 per genotype) remained in the study until they lost the ability to right themselves within 3 sec after being placed on their back, at which point they were removed from study, and categorized as “expired.” For disease progression, function was rated from score 4 (no sign of disease on any functional test) to 0 as adapted from Rouaux et al. (2007), where 3 = reduced limb extension and/or tremors upon suspension by the tail, but otherwise appears normal, 2 = deficits on functional tests (tail suspension, grip, activity, or rotarod), but no visually obvious abnormalities, 1 = visually obvious uni- or bilateral paralysis in addition to abnormalities on functional tests, 0 = loss of righting reflex, visually obvious uni- or bilateral paralysis and abnormalities on functional tests. Functional tests were as follows: (i) Grip strength: animals were held so that their hind limbs grasped the pull bar of a grip strength meter (Columbus instruments, Columbus, OH) and were pulled forward until their grip was broken. Data from five trials were normalized to body weight and expressed as compression/g of body weight. (ii) Activity test: animals were allowed to freely ambulate in a 60 × 60 cm open chamber divided into equal quadrants for 3 min. The number of times they passed into each quadrant, or reared (vertical rise) during exploration was recorded. (iii) Rotarod: mice were acclimatized on the rotarod (UgoBasile, VA, Italy) at 10 rpm (5 min) for 5 days prior to testing. For tests, mice were placed on the rotarod, and it accelerated from 10 to 54 rpm within 5 min. The time each mouse stayed on the rotarod was expressed as the latency to fall. The score shown represents the best single score from three successive rotarod trials. All functional tests were performed weekly by an investigator blinded to genotype starting from Week 8 until the animal was classified as expired.

### Statistical methods

Data are expressed as the mean ± standard error of the mean (SEM) for each group. Functional progression scores and survival data were assessed with the Kaplan–Meier statistical test. Other behavioral data were assessed with two-way ANOVA for the effect of time and score, followed by individual post hoc *t-*tests for each time point. Morphological and biochemical data were evaluated with individual *t*-tests.

## Results

### Changes in galectin-9 complement changes in galectin-3 in spinal cords of SOD1^G93A^ mice and in patients with sporadic ALS

**Figure 1:**
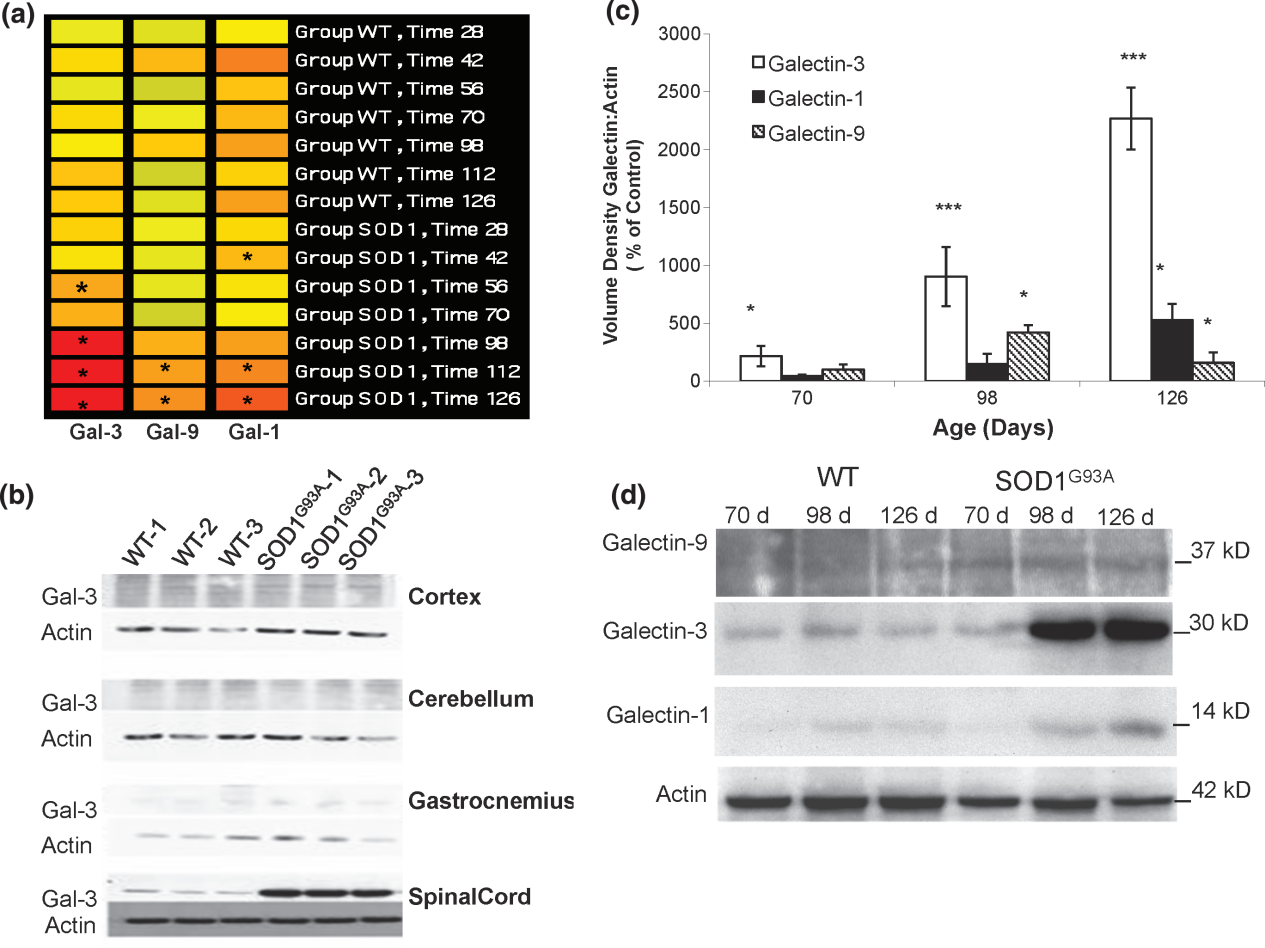
Galectins are specifically and differentially expressed in the spinal cord of mice with chronic motor neuron disease. (a) Heat map of galectin mRNA transcripts expressed over time as detected by microarray analyses of spinal cords from B6SJL SOD1 ^G93A^ versus control B6SJL (WT) mice (*n =* 3 per genotype). Each column represents the signal from a specific galectin probeset (1426808_at=galectin-3, 1421217_a_at=galectin-9, 1419573_a_at=galectin-1), and each line of bars represents mouse age (in days). Expression signal intensities are depicted as colors ranging from high (orange/warm) to low (green/cool), relative to the signal of all controls combined. Asterisks indicate significant differences from controls of equivalent age (*P* < 0.05 by Student's *t*-test comparison). In this strain, disease onset occurs at ∼98 days of age, and end-stage disease at ∼130 days of age. (b) Elevated levels of galectin-3 are restricted to the spinal cord, rather than other CNS regions or muscle. Galectin-3 protein levels were assessed with Western blots of homogenates prepared from the cortex, cerebellum, gastrocnemius muscle, and spinal cord of 126-day-old wild-type (WT) and B6SJL SOD1 ^G93A^ mice (*n* = 3 per genotype). (c) Expression of galectin-3 is elevated early in the spinal cord during motor neuron disease progression, followed by increases in galectins-9 and -1. Western blots were prepared from spinal cord homogenates of 70-, 98-, and 126-day-old B6SJL SOD1 ^G93A^ mice and age-matched strain controls (*n* = 3 per genotype). Blots were quantified, and optical densities of galectins expressed as the galectin:actin ratio as a percent of age-matched strain controls (*P < 0.05; ***P < 0.001 compared with controls by Student's *t*-test). Representative blots are shown below (d).

Galectin-1 mRNA was increased from 42 to 126 days of age, galectin-9 mRNA from 112 to 126 days of age, and galectin-3 from 56 to 126 days of age in B6SJL SOD1^G93A^ mice, compared with age-matched controls (Fig. 1a). The expression of galectin-3 protein was measured in spinal cord and other tissues at 126 days of age, when expression levels were likely to be highest. However, galectin-3 was not increased in supraspinal CNS regions (cerebral cortex, cerebellum), or in gastrocnemius muscle, which is affected in ALS and in B6SJL SOD1^G93A^ mice (Fig. 1b). Rather, it was confined to the spinal cord, where it increased from 70 to 126 days of age in B6SJL SOD1^G93A^ mice, compared with age-matched controls (∼11-fold increase from 70 to 126 days of age; Fig. 1c and d). Galectin-9 protein also increased from 98 to 126 days of age in B6SJL SOD1^G93A^ mice, whereas galectin-1 protein was not elevated until 126 days of age (Fig. 1c and d). These time points correspond to distinct functional stages in the B6SJL SOD1^G93A^ strain, where 70 days of age = asymptomatic disease, 98 days of age = onset of symptoms, and 126 days of age = end-stage/loss of righting reflex.

**Figure 2:**
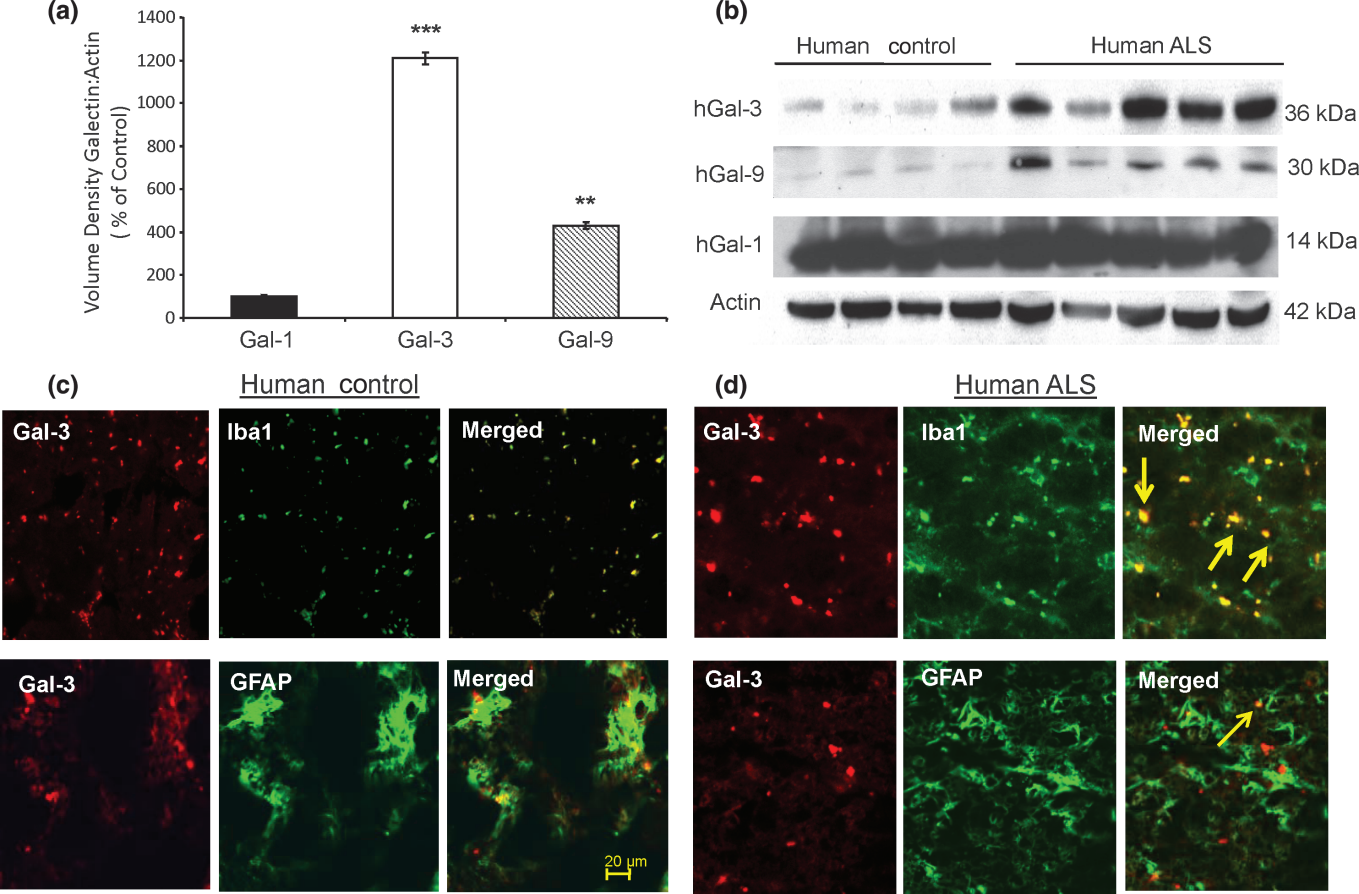
Expression of galectin-3 and -9, but not galectin-1 is elevated in patients with sporadic amyotrophic lateral sclerosis (ALS), and galectin-3 is expressed by microglia. Galectin levels in spinal cord homogenates from patients with sporadic ALS (*n =* 5) were compared with human controls of approximately equal age who died from other causes (*n* = 4), via Western blotting methods. (a) Blots were quantified as optical densities of galectins expressed as the galectin:actin ratio as a percent of optical densities from controls (**P < 0.01 or ***P < 0.001 by Student's *t*-test comparisons). (b) Images of Western blots quantified in (a). (c and d) Galectin-3 is primarily expressed by microglia in spinal cords from patients with sporadic ALS. Immunocytochemistry of transverse sections of human spinal cords from control subjects (c) and from subjects with sporadic ALS (d) is shown with double staining for galectin-3 (red) and cellular marker proteins (green). In subjects with ALS, galectin-3 predominantly colocalized with microglia cell marker Iba1 (merged-top row; thick arrows/yellow), rather than with the astrocytic specific marker GFAP (merged-bottom row, thin arrow/yellow).

Galectin-3 protein expression was also ∼12-fold greater in spinal cords from patients with sporadic ALS, relative to age-matched controls who died from other causes (Fig. 2a and b). Galectin-9 protein was approximately 4-fold higher in spinal cords from patients with ALS, whereas galectin-1 was not significantly altered.

**Figure 3:**
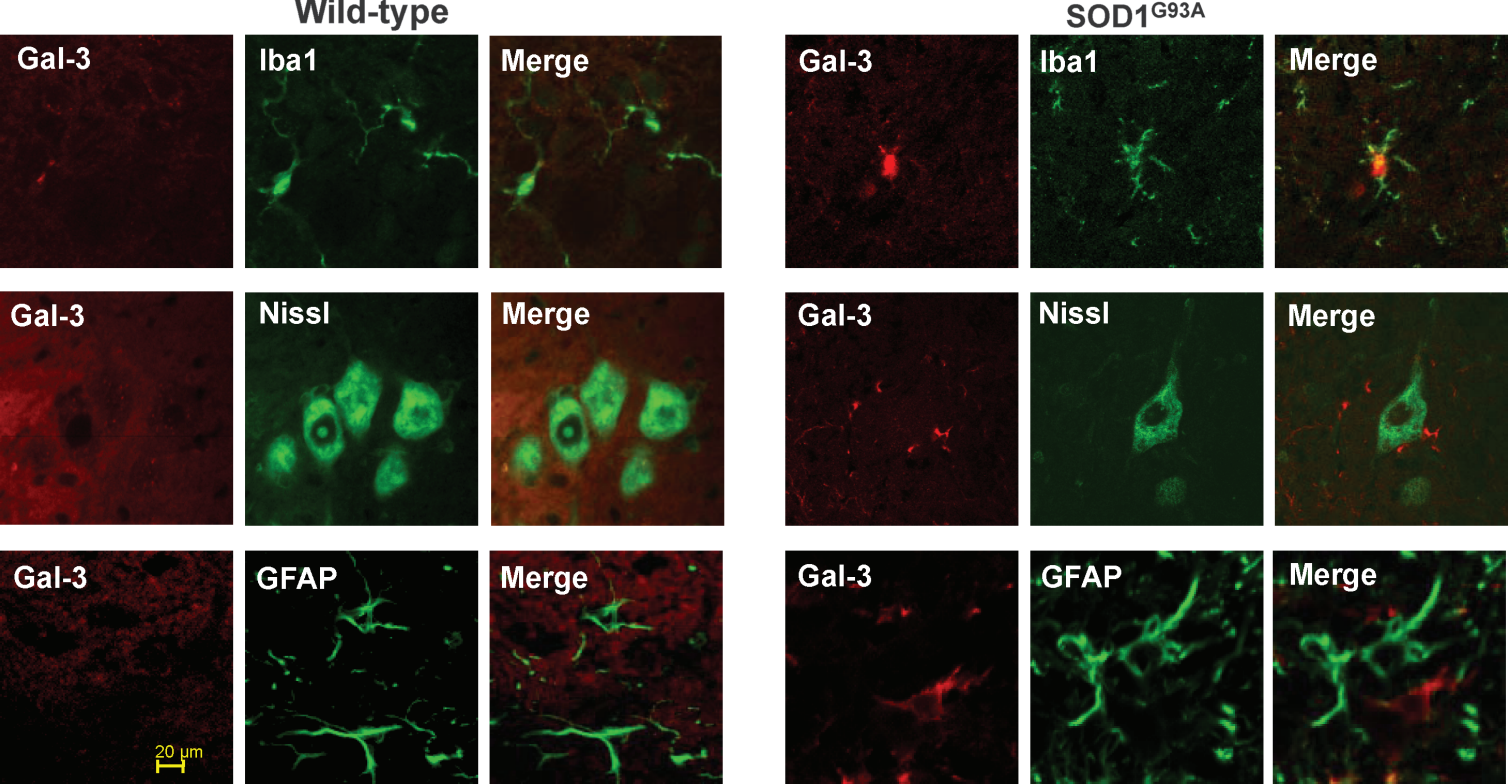
Galectin-3 is primarily expressed by microglia and some astrocytes in the B6SJL SOD1 ^G93A^ spinal cord. Representative images of transverse sections of the lumbar spinal cord from 90-day-old B6SJL SOD1 ^G93A^ transgenic mice and aged-matched WT controls were colabeled with antibodies for galectin-3 (red), and with a marker for microglia (Iba1; green; row 1), with an FITC-Nissl fluorescent stain for neurons, or with a marker for astrocytes (GFAP; green; row 3). Individual and merged images from the same field are presented.

To determine which cells express galectin-3, lumbar spinal cord sections from patients with ALS or B6SJL SOD1^G93A^ mice were double stained with antibodies to galectin-3 and several cell-type specific marker proteins and visualized with a fluorescent microscope. Galectin-3 was primarily expressed by microglia (Iba1-microglial marker, thick arrows), was also occasionally observed in astrocytes (GFAP-astrocyte marker, thin arrows; Fig. 2c and d), but was not typically expressed in neurons. Galectin-3 was also primarily expressed in microglia in B6 SJL SOD1^G93A^ mice, at 90 (Fig. 3) and 130 days of age (data not shown).

### Deletion of galectin-3 accelerates disease progression and weight loss, diminishes motor function, and reduces life span of SOD1 ^G93A^ mice

To avoid confounding effects of background strain, C57BL6 SOD1^G93A^ mice were used to create the SOD1^G93A^ galectin-3 knock-outs. SOD1^G93A^ mice on the C57BL6 background have a longer life span than SOD1^G93A^ mice on the B6SJL strain, with end-stage disease (50% survival) typically occurring at around 160 versus 130 days of age, respectively (Gurney et al. 1994).

**Figure 4:**
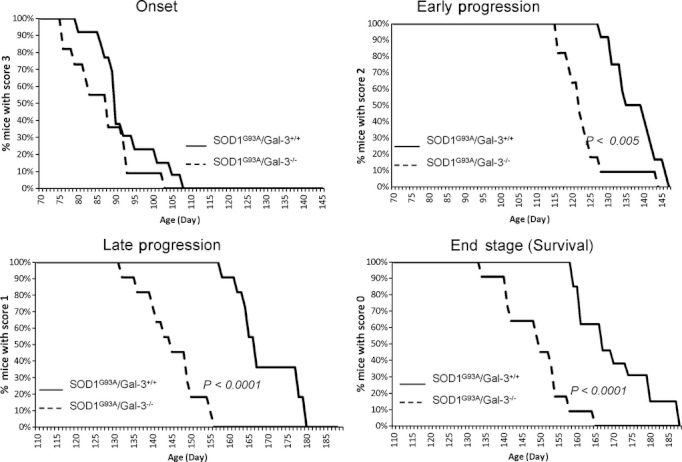
Progression of motor neuron disease is more rapid and life span is decreased in C57BL6 SOD1^G93A^/Gal-3^−/−^ mice (*n =* 12) compared with C57BL6 SOD1^G93A^/Gal-3^+/+^ controls (*n =* 12). A neurological assessment rating scale from score 4 (no visible sign of disease) to 0 was used to assess disease progression. Each graph represents the % of mice with scores 3 through 0 for each genotype over time, where 3 = altered hind limb extension/tremors on suspension by the tail (aka failed “splay” test), but otherwise visually normal, 2 = deficits on “splay” test and additional functional tests (grip, activity, or rotarod), but no visually obvious abnormalities; 1 = deficits on all tests and visually observable uni- or bilateral paralysis; and 0 = deficits on all tests, visually obvious uni- or bilateral paralysis, and fails righting reflex test. The *P*-values shown represent the Kaplan–Meier statistical analysis result for comparisons of the genotypes at each disease stage/score as indicated on the *y*-axis. Animals that received a score of 0 were removed from the study and considered expired, and thus, the graph of mice with score = 0 is equivalent to a Kaplan–Meier survival curve.

**Figure 5:**
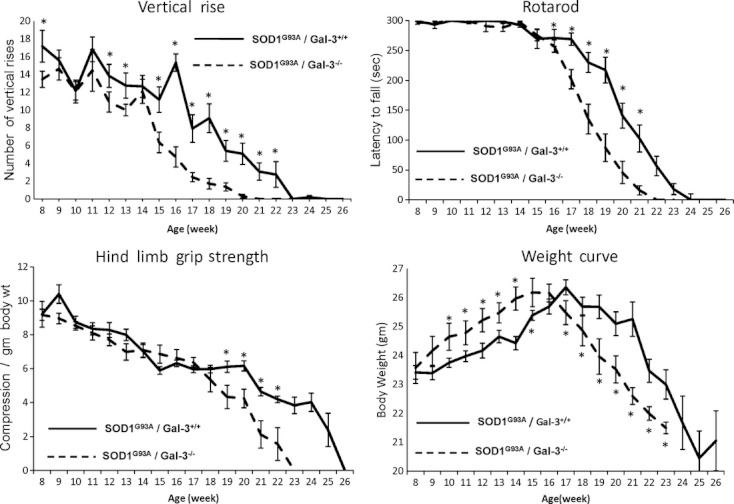
The C57BL6 SOD1^G93A^/Gal-3^−/−^ phenotype (*n =* 12) displays greater functional impairment and weight loss than the C57BL6 SOD1^G93A^/Gal-3^+/+^ cohort (*n =* 12) during disease progression. Mean ± SEM scores of C57BL6 SOD1^G93A^/Gal-3^−/−^ or C57BL6 SOD1^G93A^/Gal-3^+/+^ control mice are graphed over time for individual tests of motor function or body weight. The vertical rise score reflects the number of times animals reared up onto their hind limbs to explore during 3 min in an open field chamber. Rotarod and grip strength tests are detailed in Methods. For all, *P < 0.05 by two-way ANOVA followed by individual post hoc *t*-tests for each time point.

Deletion of galectin-3 in SOD1^G93A^ diseased (C57BL6 SOD1^G93A^/Gal-3 ^−/−^) mice did not alter the date of the first appearance of a neurobehavioral defect (onset), but it hastened progression to more severely impaired disease stages (Fig. 4). In addition, the survival of C57BL6 SOD1^G93A^/Gal-3^−/−^ mice was reduced by 25 days compared with C57BL6 SOD1^G93A^/Gal-3^+/+^ controls. C57BL6 SOD1^G93A^/Gal-3^−/−^ mice also scored poorly compared with C57BL6 SOD1^G93A^/Gal-3^+/+^ mice on the standard functional tests (vertical rise, rotarod, hind limb grip strength; Fig. 5). Prior to disease onset, C57BL6 SOD1^G93A^/Gal-3^−/−^ mice had increased body weight compared with C57BL6 SOD1^G93A^/Gal-3^+/+^ mice (Fig. 5). However, starting at week 17, C57BL6 SOD1^G93A^/Gal-3^−/−^ mice had an acceleration of weight loss relative to C57BL6 SOD1^G93A^/Gal-3^+/+^ cohorts.

**Figure 6:**
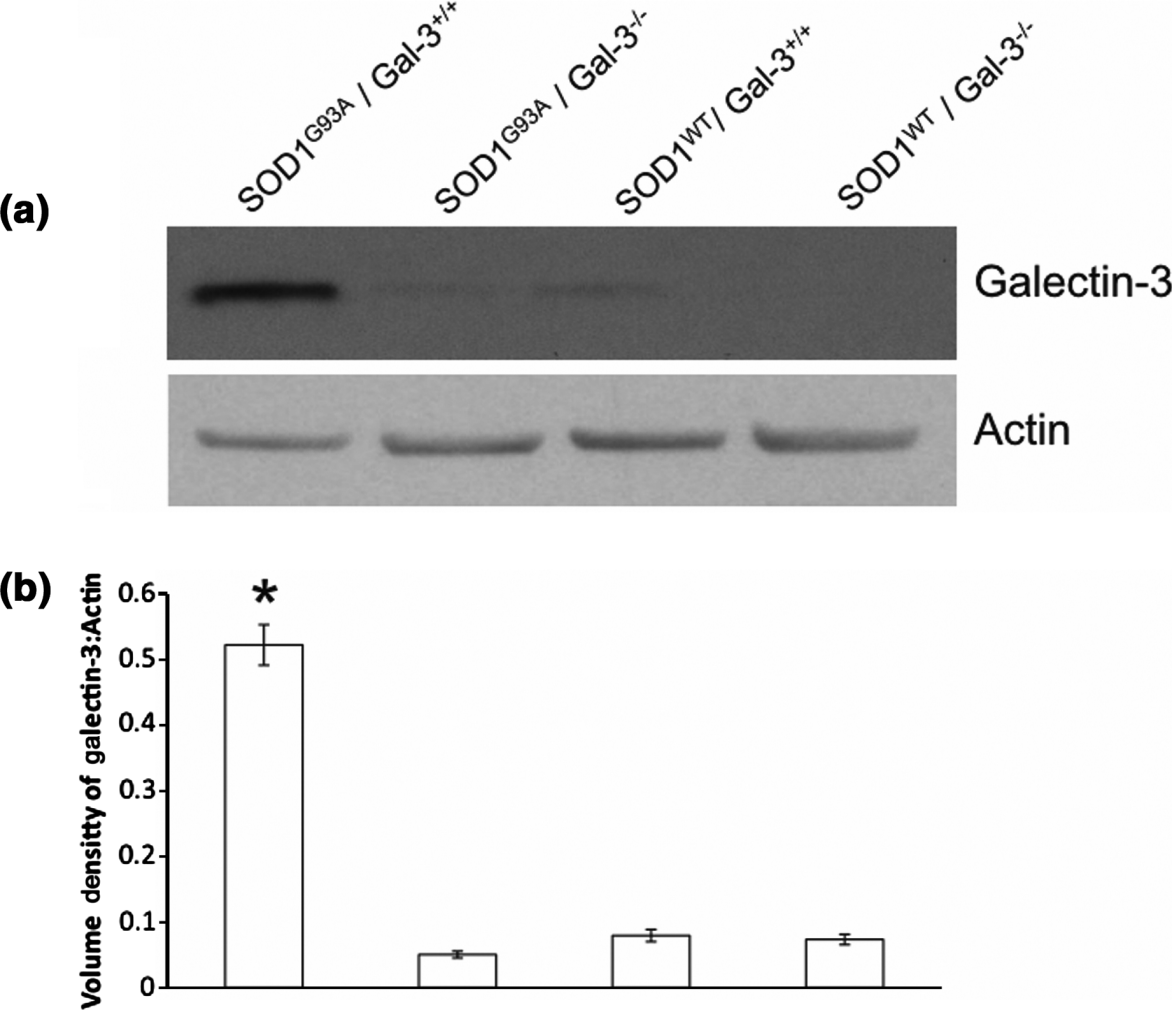
Verification that galectin-3 is increased with SOD1^G93A^ mutation on the C57BL6 background strain and is abolished in the SOD1^G93A^/Gal-3^−/−^ phenotype. Western blots were prepared from spinal cord homogenates obtained at the end stage of disease (126 days of age) in SOD1^G93A^/Gal-3^+/+^, SOD1^G93A^/Gal-3^−/−^, SOD1^WT^/Gal-3^+/+^, and SOD1^wT^/Gal-3^−/−^ (*n =* 3 per genotype) animals. A representative blot is shown in (a), with the mean ± SEM optical density of galectin-3 signal normalized to actin graphed for all blots/groups shown in (b) *P < 0.005 versus SOD1^G93A^/Gal-3 ^−/−^ by Student's *t*-test comparison.

Western blots confirmed elevation of galectin-3 in the C57BL6 SOD1G93A/Gal-3^+/+^ strain, and that deletion of galectin-3 abolished galectin-3 expression (Fig. 6). Galectin-3 was also not elevated in either C57BL6 SOD^WT^/Gal-3^+/+^ or C57BL6 SOD^WT^/Gal-3^−/−^ mice (Fig. 6), and C57BL6SOD^WT^/Gal-3^−/−^ control mice showed no evidence of functional impairment or decreased life span.

### Deletion of galectin-3 increases microglial activation, TNF-α levels, and oxidative injury in SOD1 ^G93A^ mice

**Figure 7:**
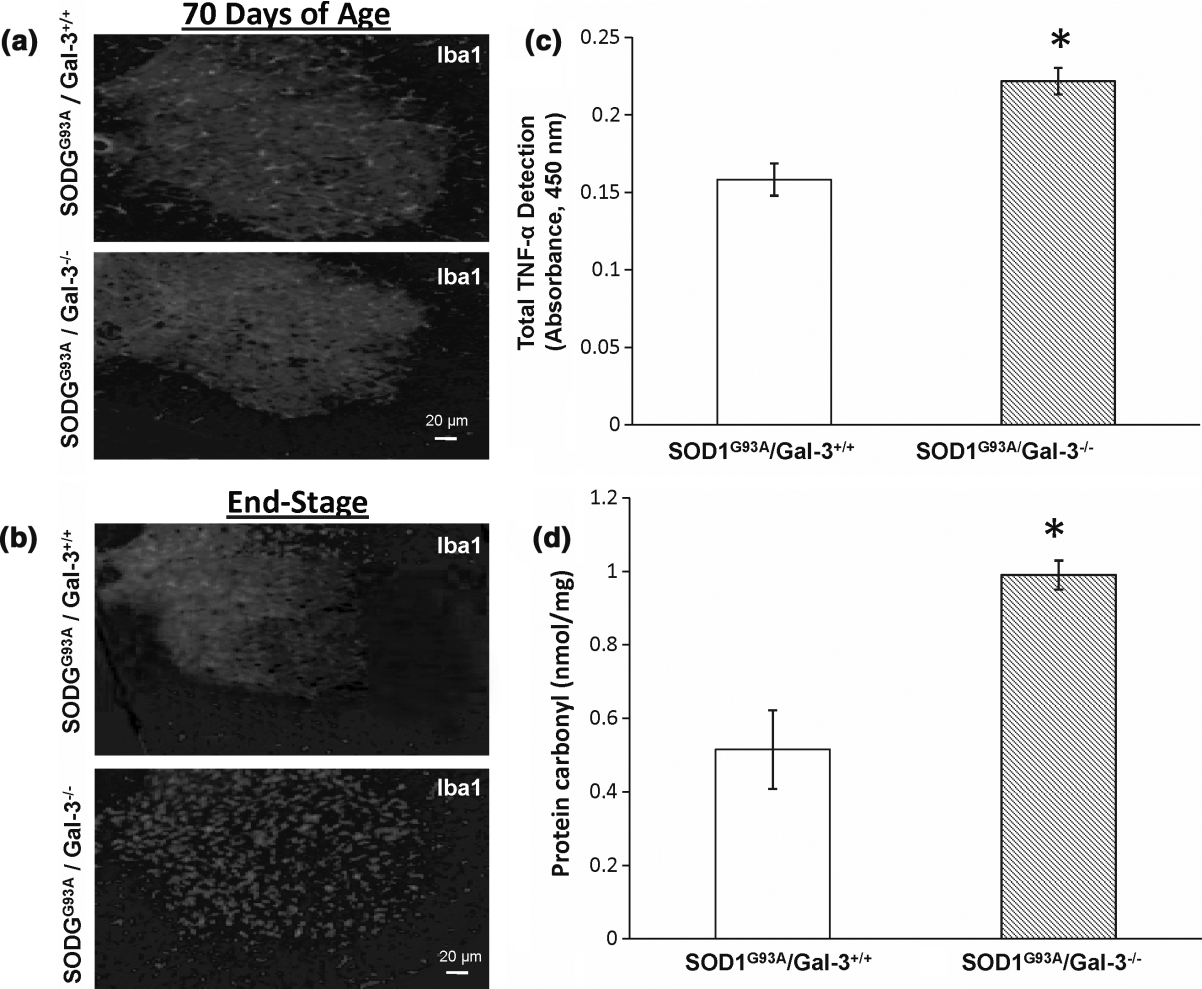
IBA1 positive cells (microglia), TNF-α, and oxidative injury are increased in C57BL6 SOD1^G93A^/Gal-3^−/−^ mice compared with C57BL6 SOD1^G93A^/Gal-3^+/+^ controls at the end stage of motor neuron disease. Sections from either (a) 70-day-old or (b) end-stage (loss of righting reflex) lumbar spinal cords (*n* = 6 for each genotype, at each time point) were stained with anti-IBA1. Representative images are shown. (c) TNF-α and oxidative injury (d) are increased in C57BL6 SOD1^G93A^/Gal-3^−/−^ mice compared with C57BL6 SOD1^G93A^/Gal-3^+/+^ controls (*n* = 4 for each genotype) at the end stage of motor neuron disease. TNF-α and protein carbonyls (as markers of oxidative protein modification) were assessed in homogenates from lumbar spinal cords using ELISA assays (*P < 0.05 by Student's *t*-test). TNF-α levels are expressed as mean ± SEM absolute absorbance values. Carbonyls were derived from a standard curve prepared from reduced oxidized BSA standards and expressed as mean ± SEM nmol/pg protein.

Sections were immunostained for the microglial marker Iba1 and positive cells visualized in mice at 70 days of age and at end stage of disease (Fig. 7a and b). There were slightly more Iba1 positive cells in C57BL6 SOD1^G93A^/Gal-3^+/+^ controls versus C57BL6 SOD1^G93A^/Gal-3^−/−^ mice at 70 days of age, but the difference was not marked. However, Iba1 positive cells were increased at end stage relative to 70 days of age for both genotypes – and there was a marked increase in Iba1 positive cells in C57BL6 SOD1^G93A^/Gal-3^−/−^ compared with age-matched C57BL6 SOD1^G93A^/Gal-3^+/+^ controls at this time. To determine whether activation of microglia may correlate with enhanced inflammation, the level of TNF-α in the spinal cord of diseased mice was measured at end stage of disease. TNF-α in the lumbar spinal cord of C57BL6 SOD1^G93A^/Gal-3^−/−^ mice was higher than in C57BL6 SOD1^G93A^/Gal-3^+/+^ controls (Fig. 7c). To evaluate whether the observed exacerbation of neuroinflammation in C57BL6 SOD1^G93A^/Gal-3^−/−^ mice was associated with oxidative damage, protein carbonyls were quantified. Protein carbonyls are formed by free radical mediated amino acid (proline, arginine, lysine, and threonine) modification. Total protein carbonyl content was nearly doubled in C57BL6 SOD1^G93A^/Gal-3^−/−^mice compared with C57BL6 SOD1^G93A^/Gal-3^+/+^ controls (Fig. 7d).

## Discussion

The glial-derived neuroinflammatory microenvironment can significantly alter the progression of ALS. The present data show progressive increases in expression of several galectins in the spinal cord of the SOD1^G93A^ murine model of ALS. Galectin-3 protein expression was initially elevated at the presymptomatic stage of disease (10 weeks), and increased through end stage. It was also confined to the spinal cord and thus appears to be specifically associated with the ongoing degenerative process there. The primary source of galectin-3 in the SOD1^G93A^ mouse and in humans with ALS was activated microglia, as it was only occasionally observed in astrocytes or neurons. Expression of galectin-9 increased after symptoms appeared, and galectin-1 increased only at the end stage of disease; therefore, the expression of these galectins represents a later event in disease progression. Further, only galectin(s)-3 and galectin-9 were increased in human postmortem spinal cords of patients with sporadic ALS.

Our study using Western blots did not detect galectin-1 in patients with ALS, but others have localized it to neurofilamentous lesions associated with the disease (Kato et al. 2001). The discrepancy may reflect methodological differences, as the Western blots used here may not detect subtle, localized changes that are visualized with histology. Notably, intramuscular administration of oxidized galectin-1 improved neurological function and extended survival in a mutant SOD1 mouse model of ALS (Chang-Hong et al. 2005).

To our knowledge, the present data are the first to identify elevated galectin-9 associated with motor neuron disease and ALS. In vitro, galectin-9 is expressed by astrocytes stimulated with IL-1β (Yoshida et al. 2001), and this effect is abolished by dexamethasone, suggesting that galectin-9 is produced as part of neuroinflammation induced by pro-inflammatory cytokines. However, we speculate that it may potentially reduce inflammation, as galectin-9 is a ligand for T-cell immunoglobulin mucin-(TIM)-3, and activation of the galectin-9-TIM-3 pathway suppresses Th1 immune responses (Zhu et al. 2005). Galectin-9 also reduces secretion of TNF-α and IL-1β and increases IL-10 production in stimulated peritoneal macrophages (Arikawa et al. 2009). Additional studies are needed to determine the functional role of galectin-9 in ALS.

In agreement with previous reports (Yamanaka et al. 2008; Saxena et al. 2009; Hossiani et al. 2011), our data implicate microglia as the major source of galectin-3 associated with chronic motor neurodegeneration. However, others noted galectin-3 (but not galectin-1 or -9) mRNA increased in motor neurons from late-stage disease (∼17 weeks) SOD1^G93A^ mice (Ferraiuolo et al. 2007). Expression profiling methods also detected galectin-1 and -3 mRNA in spinal cord and skeletal muscle of paralyzed SOD1^G86R^ mice at 15 weeks of age (Gonzalez de Aguilar et al. 2008). Increased galectin-3 protein was recently observed in spinal cords from SOD1^G93A^ mice and patients with ALS (Zhou et al. 2010), where it was also observed in CSF, and suggested that it may be a potential clinical biomarker of motor neuron disease. The present data extend such observations by showing that the initial elevation of galectin-3 occurs even in the presymptomatic stage of disease, and that it increases further from that point.

We generated C57BL6 SOD1^G93A^/Gal-3^−/−^ knock-out transgenic mice, to evaluate the effect of galectin-3 deletion on the diseased phenotype. Mice with the galectin-3 deletion on the pure (undiseased) C57BL6 background have been characterized as viable and fertile, with the same body and organ weights as galectin-3^+/+^ cohorts (Hsu et al. 2000). In initial observations, they displayed no overt behavioral defects, no abnormalities in blood chemistry or cell counts, and histological evaluation revealed no gross abnormalities of major organs, including brain. Indeed, C57BL6 SOD^WT^/Gal-3^−/−^ mice did not perform significantly different from C57BL6 SOD^WT^/Gal-3^+/+^ animals at any point in the present study. C57BL6 galectin-3^−/−^ mice <90 days of age also performed identically to controls on locomotor, hole-board, or inverted screen tests in another recent study; however, they displayed an increased percentage of open arm entries in a plus-maze test, suggesting reduced anxiety (Pasquini et al. 2011). Moreover, histological analysis revealed defects in myelin structure and oligodendrocyte differentiation. Thus, these features of the C57BL6 galectin-3^−/−^ phenotype may be present in SOD1^G93A^/Gal-3^−/−^ transgenics, although we did not perform histology to verify that, so this issue remains unresolved.

Galectin-3 deletion did not alter disease onset, though SOD1^G93A^/Gal-3^−/−^mice progressed faster through all stages of disease and expired, on average, 25 days earlier than their SOD1^G93A^/Gal-3^+/+^ cohorts. SOD1^G93A^/Gal-3^−/−^ mice also scored significantly worse on all functional tests relative to SOD1^G93A^/Gal-3^+/+^ mice, indicating that the SOD1^G93A^/Gal-3^−/−^ phenotype was severely impaired, and suggesting that galectin-3 deletion may have increased neurodegeneration. Deletion of galectin-3 was also associated with an increase in microglia, and elevated levels of TNF-α and protein carbonyls, a marker of oxidative injury.

Microglial number and phenotype significantly influence the progression of motor neuron disease. Increases in wild-type (non-SOD1^G93A^) microglia reduced neurodegeneration slowed disease progression and increased survival of SOD1^G93A^ mice, supporting a generally protective role for healthy microglia against the disease process (Beers et al. 2006). Alternatively, selective reduction of SOD1 overexpression within diseased microglia increased survival time, particularly at the end stage of disease (Boillee et al. 2006; Wang et al. 2010), suggesting that mSOD1 imparts neurotoxic properties to microglia that contribute to neurodegeneration. Indeed, SOD1^G03A^ microglia display an inflammatory phenotype characterized by elevations in TNF and other inflammatory molecules (Sargsyan et al. 2009). Thus, the presence of more SOD1^G93A^ microglial cells in the SOD1^G93A^/Gal-3^−/−^ cohort may have increased the neurotoxic/pro-inflammatory load on neurons, and accelerated disease progression. Also possible, is that deletion of galectin-3 further enhanced the already neurotoxic properties of the mSOD1^G93A^ microglia. This is indirectly supported by data showing that a 50% reduction in the number of proliferating galectin-3/Mac-2^+^ mutant (mSOD1) microglia in CD11b-TK^mut−30^, SOD1^G93A^ doubly transgenic mice had no effect on neurodegeneration (Gowing et al. 2008).

Increased protein carbonyls correlate with an upregulation of TNF-α transcripts at an early stage of disease in SOD1^G93A^ mice (Hensley et al. 2006). TNF-α elevation also correlates with disease progression in the mouse, and TNF-α is increased in serum from ALS patients (Poloni et al. 2000; Hensley et al. 2003). Although the precise role of TNF in disease progression is unclear (Gowing et al. 2006), it has many pro-inflammatory effects, including activation and stimulation of free radical release from microglia, as well as potential synergisms with other pathogenic factors involved in the disease (McGeer and McGeer 2002; Mir et al. 2009). While the present studies do not address any direct relationship between TNF-α and oxidative injury, the observation that both were increased in SOD1^G93A^/Gal-3^−/−^ mice supports that neuroinflammation was exacerbated in the absence of galectin-3.

Though not always consistent (Doverhag et al. 2010), some evidence suggests a protective role for galectin-3^+^/Mac-2 expressing microglia, at least in models of acute neurodegeneration (Lalancette-Hebert et al. 2007). Such protection is associated with transition of microglia to a galectin-3 positive alternative activation phenotype (M2) that expresses increased levels of IGF-1 and a type Th2 immune bias (Ohtaki et al. 2008). Importantly, deletion of galectin-3 from macrophages renders them unable to assume the M2 phenotype (MacKinnon et al. 2008). In addition, galectin-3 is known to be involved in a variety of physiological phenomena associated with alternative activation, as it promotes wound healing, angiogenesis, and growth and proliferation of neural progenitors (Pesheva et al. 1998; Cao et al. 2002; Yan et al. 2009). Considered together with our data, these observations support the speculation that deletion of galectin-3 may have eliminated the trophic and reparative effects of an alternatively activated microglial phenotype in the SOD1^G93A^/Gal-3^−/−^cohort and that an activated pro-inflammatory (M1) microglial phenotype may have predominated in the SOD1^G93A^/Gal-3^−/−^ microenvironment. Nevertheless, any such effect on neuroinflammation may be conditionally dependent, as galectin-3 depletion reduced inflammation and the severity of experimental autoimmune encephalitis (Jiang et al. 2009), and reduced pain due to macrophage invasion in herpes zoster induced allodynia (Takasaki et al. 2012).

Although this study focuses on neuroinflammation, galectin-3 is pleiotropic and may modulate other factors involved in motor neuron disease. For example, intracellular galectin-3 directly influences necrotic and apoptotic cell death pathways, as well as autophagy (Yu et al. 2002; Mok et al. 2007). Galectin-3 is also an advanced glycation end-product (AGE) receptor (RAGE) that targets AGE for lysosomal degradation and removal (Pricci et al. 2000). As AGE are a source of inflammation and oxidative injury both in ALS and mouse models of ALS (Kato et al. 2000), deletion of galectin-3 may enhance neurodegeneration due to AGE accumulation. Glycoprotein receptors for T cells, trophic factors (EGF, IGF), and cytokines with the consensus sequence (N-X-S/T) also contain *N*-acetyllactosamines, which are preferred binding substrates for extracellular galectin-3 (Rabinovich et al. 2007). Galectin-3 forms cross-linked lattices with these residues that alter downstream cell signaling and inflammatory pathways (reviewed in Boscher et al. 2011), and such interactions would likely be reduced by galectin-3 deletion. In addition, as noted previously, galectin-3 deficient mice have defects in myelin integrity and reduced oligodendrocyte differentiation, and these may have influenced functional outcome both in controls and in the SOD1^G93A^ transgenics (Pasquini et al. 2011). Galectin-3 may also influence neuroblast migration in the developing brain (Comte et al. 2011). Although we have not addressed any of these mechanisms in the present report, they may have contributed to our observations.

Finally, although it is interesting to speculate that there may be differences in galectin-3 expression and related inflammatory changes between C57/B6 G93A SOD1 and SJL SOD1^G93A^ mice that contribute to the overall significantly faster disease progression in the SJL SOD1^G93A^ strain, it is worth noting that the pure SJL/J strain has reduced levels of dysferlin, altered macrophage activity, early functional impairment, and indeed is used as a model of limb girdle muscular dystrophy type 2B (Rayavarapu et al. 2010). If and how these traits may potentially contribute to SJL × C57/B6 strain-specific onset/progression and drug response characteristics in hSOD1 transgenics is unclear.

In conclusion, the present data indicate that galectin(s)-3 and -9 are increased in ALS and an SOD1^G93A^ mouse model of ALS. Galectin-3 is primarily expressed by microglia in the SOD1^G93A^ mouse model of ALS, and in humans with ALS. Deletion of galectin-3 in the SOD1^G93A^ mouse resulted in rapid disease progression, and increases in microglia, TNF-α, and oxidative injury, compared with galectin-3 expressing SOD1^G93A^ diseased controls. Thus, endogenous production of galectin-3 by microglia may, at least in part, serve to limit neuroinflammation and disease progression during chronic motor neurodegenerative disease.
